# Nilotinib for the Frontline Treatment of Chronic Myeloid Leukemia Carrying the p230 Transcript: Dream or Reality?

**DOI:** 10.3389/fonc.2014.00017

**Published:** 2014-02-03

**Authors:** Maurizio Capuozzo, Alessandro Ottaiano, Eduardo Nava, Stefania Cascone, Claudia Cinque, Adriano Vercellone, Corinne Scognamiglio, Emilia Palumbo, Rosario V. Iaffaioli

**Affiliations:** ^1^Department of Pharmacy, Local Sanitary Agency (LSA) Naples 3 South, Naples, Italy; ^2^Department of Colorectal Oncology, National Cancer Institute “G. Pascale” Foundation, Naples, Italy; ^3^Local Sanitary Agency (LSA) Naples 1 Center, Naples, Italy

**Keywords:** nilotinib, chronic myeloid leukemia, e19a2 rearrangement, tyrosine kinase inhibitors, chromosomal translocation

The therapeutic landscape of chronic myeloid leukemia (CML) has changed dramatically in the last decade (1). In particular, the availability of imatinib mesylate, a tyrosine kinase inhibitor (TKI) targeting BCR–ABL, has led to profound and durable remissions in the majority of patients. However, a significant proportion of patients either present with primary resistance to imatinib or develop secondary resistance sooner or later during treatment. Therefore, second-generation TKIs have been introduced, including nilotinib. In fact, genetic mutations in BCR–ABL result in several possible changes and lead to subsequent TKI resistance (2). It is known that the most cases of CML are characterized by a BCR–ABL fusion protein originating from the t(9;22) chromosomal translocation. The exact breakpoint of the translocation and the molecular weight of the resulting fusion gene protein vary. Mostly, the breakpoint on chromosome 22, falling in the so-called major breakpoint cluster region between exons 13 and 14 of the BCR gene (M-bcr), leads to a hybrid BCR–ABL mRNA with a b2a2 or b3a2 junction, which encodes a p210 fusion protein associated with the underlying mechanism, in the chronic phase of CML (3). Cases of CML with breakpoints in other regions are seen in other regions, namely minor (m-bcr) and micro (μ-bcr) bcr region, but to date, these cases are few in number. In particular, the μ-bcr breakpoint connects exon 19 of BCR with ABL (exon 2 of which is the joining point in all three cases), giving rise to the e19a2 transcript corresponding to the p230 fusion protein. The e19a2 rearrangement was detected in patients with typical CML or in those with a rather aggressive clinical course (4). However, our experience has given us great hopes for the future. In fact, a 39-year-old male patient was diagnosed with chronic phase CML in March 2012. The examination of his peripheral blood showed a hemoglobin level of 12.6 g/dL, mean corpuscular volume of 112 fL, white blood cell count of 58 × 10^9^/L (with differential counts neutrophils 41%, lymphocytes 0%, monocytes 0%, eosinophils 0.5%, basophils 0.9%, myeloblasts 2%, myelocytes 29%, metamyelocytes 20%), and a platelet count of 212 × 10^9^/L. After bone marrow aspirate, the cytogenetic analysis revealed 46, XY, t(9;22)(q34;q11.2) karyotype in 100% of metaphase. The quantitative reverse transcription polymerase chain reaction (RQ-PCR) detected the sole presence of the e19a2 transcript. After starting frontline treatment with nilotinib 600 mg/day (April 2012), the patient achieved a complete cytogenetic response (after 4 months) and complete molecular response (CMR), defined as the absence of detectable BCR–ABL transcript by RQ-PCR (after 6 months). Currently, 18 months after the start of the treatment, the patient continues to take 600 mg nilotinib per day and CMR is confirmed. We have found only two reporting data on nilotinib treatment in patients with the e19a2 rearrangement (5, 6). Interestingly, the use of nilotinib has induced a fast CMR also in our patient who maintained a CMR for over 1 year until today. It is mandatory to improve our ability to predict outcomes in our patients using *ad hoc* molecular tests (DNA sequencing and gene expression profiling), in order to offer the optimal strategy to individual patients. In our experience, we recommend the use of nilotinib as frontline agent for the treatment of this CML variant, according to the evidence of a deep and rapid molecular response obtained in our patient.

In addition, accurate clinicobiological evaluation, an evidence-based approach, and identification of potential biomarkers are definitely warranted to delineate the best approach in a given case.
